# Follow-up study of high-dose praziquantel therapy for cerebral sparganosis

**DOI:** 10.1371/journal.pntd.0007018

**Published:** 2019-01-14

**Authors:** Peng Zhang, Yang Zou, Feng-Xia Yu, Zheng Wang, Han Lv, Xue-Huan Liu, He-Yu Ding, Ting-Ting Zhang, Peng-Fei Zhao, Hong-Xia Yin, Zheng-Han Yang, Zhen-Chang Wang

**Affiliations:** 1 Department of Radiology, Beijing Friendship Hospital, Capital Medical University, Beijing, China; 2 Beijing Institute of Tropical Medicine, Beijing Friendship Hospital, Beijing Key Laboratory for Prevention and Treatment of Tropical Diseases, Capital Medical University, Beijing, China; 3 Medical Imaging Center, Beijing Tongren Hospital, Capital Medical University, Beijing, China; University of Melbourne, AUSTRALIA

## Abstract

**Background:**

Cerebral sparganosis is the most serious complication of human sparganosis. Currently, there is no standard for the treatment of inoperable patients. Conventional-dose praziquantel therapy is the most reported treatment. However, the therapeutic outcomes are not very effective. High-dose praziquantel therapy is a useful therapeutic choice for many parasitic diseases that is well tolerated by patients, but it has not been sufficiently evaluated for cerebral sparganosis. This study aims to observe the prognoses following high-dose praziquantel therapy in inoperable patients and the roles of MRI and peripheral eosinophil absolute counts during follow-up.

**Methodology:**

Baseline and follow-up epidemiological, clinical, radiological and therapeutic data related to 10 inoperable patients with cerebral sparganosis that were treated with repeated courses of high-dose praziquantel therapy, with each course consisting of 25 mg/kg thrice daily for 10 days were assessed, followed by analyses of the prognoses, MRI findings and peripheral eosinophil absolute counts.

**Principal findings:**

Baseline clinical data: the clinical symptoms recorded included seizures, hemiparesis, headache, vomiting and altered mental status. Peripheral blood eosinophilia was found in 3 patients. The baseline radiological findings were as follows. Motile lesions were observed in 10 patients, including aggregated ring-like enhancements, tunnel signs, serpiginous and irregular enhancements. Nine of the 10 patients had varying degrees of white matter degeneration, cortical atrophy and ipsilateral ventricle dilation. The follow-up clinical data were as follows. Clinical symptom relief was found in 8 patients, symptoms were eliminated in 1 patient, and symptoms showed no change from baseline in 1 patient. Peripheral blood eosinophilia was found in 2 patients. The follow-up radiological findings were as follows. Motile lesions that were transformed into stable, chronic lesions were found in 8 patients, and motile lesions that were eliminated completely were found in 2 patients.

**Conclusions:**

High-dose praziquantel therapy for cerebral sparganosis is effective. The radiological outcomes of motile lesions are an important indicator during the treatment process, especially during follow-ups after clinical symptoms have improved. Peripheral eosinophil absolute counts cannot be used as an effective prognostic indicator.

## Author summary

Sparganosis is a rare parasitic disease with a high prevalence in East Asia. Because of limited radiological technology and clinical experience, the prevalence of cerebral sparganosis is likely underestimated in developing countries. Cerebral sparganosis is the most serious complication of human sparganosis. Currently, it is generally believed that the most effective treatment for cerebral sparganosis is surgical treatment. However, the choice of treatment is a challenge for inoperable patients, including those with multifocal lesions or lesions in deep structures or important functional areas and those refusing invasive treatment due to personal willingness. Currently, there is no standard for the treatment of inoperable patients. In addition, anthelmintic treatment for sparganosis has rarely been reported in the literature. High-dose praziquantel therapy is a useful therapeutic choice for many cerebral parasitic diseases, including neurocysticercosis, and is well tolerated for patients, but it has not been sufficiently evaluated for the treatment of cerebral sparganosis. This study aims to describe the clinical, radiological and therapeutic data following high-dose praziquantel therapy for ten inoperable patients. All patients reached clinical cure after one to five courses. These results suggest that high-dose praziquantel therapy for cerebral sparganosis could achieve favorable outcomes and that MRI plays an important role in follow-up, especially when clinical symptoms have improved.

## Introduction

Sparganosis is a rare parasitic disease caused by an infection by the second-stage larvae of *Spirometra mansoni*, also called “sparganum”. There is a higher prevalence of this disease in East Asian countries, such as China, Thailand, Korea and Japan, although sparganosis also occurs occasionally in Europe, North Americaand South America[1–8].

Although a series of cases have been reported in recent years, the prevalence of sparganosis is still likely underestimated. Because of limited computed tomography (CT) and magnetic resonance imaging (MRI) technology and limited clinical experience, many cases in developing countries are likely unreported[9]. Sparganum can be found in many soft tissues of the human body, such as subcutaneous tissue, muscles, breast tissue, peritoneum and pleura, which are primarily composed of connective tissue[10–12]. Cerebral sparganosis is defined as a brain infection of sparganum, which is the most serious complication of human sparganosis. By combining CT and MRI data, epidemiological history, enzyme-linked immunosorbent assay (ELISA) testing for parasitic antibodies and stereotactic biopsy, most cases of sparganosis can be diagnosed[13]. Currently, it is generally believed that the most effective treatment for cerebral sparganosis is the surgical removal of the sparganum[2, 3, 14]. However, the choice of treatment is a challenge for inoperable patients, including those with multifocal and surgical contraindicated lesions. Currently, there is no standard for the treatment of inoperable patients, including anthelmintic treatments. High-dose praziquantel therapy has been applied to cysticercosis, and preliminary results were encouraging[15, 16]. However, although several case reports in recent years have shown that high-dose praziquantel therapy for cerebral sparganosis has favorable outcomes, there is still insufficient clinical experience, and no systematic follow-up studies have been performed in inoperable patients[17, 18]. The purpose of this study is to observe the prognoses following high-dose praziquantel therapy for cerebral sparganosis, to investigate the roles of MRI and peripheral eosinophil absolute counts in therapy and to further provide clinical treatment, follow-up evaluation and management experience for cerebral sparganosis, especially for inoperable patients.

## Materials and Methods

The retrospective study was approved by the Ethical Committee of Beijing Friendship Hospital, Beijing, China (2018-P2-074-01). The patients’ data were anonymized/deidentified to protect the patients’ privacy/confidentiality. This study was conducted in accordance with the Declaration of Helsinki.

### Subjects

The medical records and radiological data for 10 patients, who were registered between 2013 and 2017, were reviewed retrospectively. These patients included 7 males and 3 females, aged between 7 and 45 years old, with a mean age of 20.3 years old. All patients were diagnosed based on a collection of findings, including ingestion history associated with sparganum, clinical manifestations, positive ELISA results of a sparganum antibody in serum and at least two MRI features of cerebral sparganosis (aggregated ring-like enhancements, serpiginous enhancements, irregular enhancements, tunnel signs or migration signs). The presence of antibodies against *S*. *mansoni* in serum was assessed by ELISA kits (# JL 0702193, Jianlun Biology Technology Co., LTD, Guangzhou, P.R. China). All patients underwent stool examinations. All patients were inoperable due to the following reasons. First, there were multifocal lesions and functional deficiencies. Second, the lesions were in areas where operation was contraindicated. Third, the patients refused invasive treatment.

### Treatment

All patients were treated with high-dose praziquantel. The high-dose praziquantel treatment consisted of daily praziquantel at a dose of 75 mg per kilogram of body weight, which was administered in three divided doses, for 10 days. Dexamethasone was administered if symptoms exacerbated with praziquantel therapy. 5 mg was given initially followed by 10 mg/day until the end of the 10 day course and then tapered with 5 mg/ for 3 days and then 4.5 mg/day for 5 days. The patients who presented with symptomatic epilepsy were treated with oxcarbazepine. Daily oxcarbazepine at a dose of 10 mg per kilogram of body weight, which was administered in two divided doses, was used to treat parasite-induced symptomatic epilepsy. The anticonvulsive treatment was slowly tapered when the seizures discontinued[1, 17, 19, 20]. Praziquantel was administered in 10 day courses every 3 months until there was clinical improvement and serial MRI studies showed disappearance of the initial enhancing lesions or replaced by stable, chronic lesions. Repeated 10 day courses were also given if new symptoms recurred. At least 3 follow-up MRI assessments were performed for each patient. The first MRI assessment was the baseline examination, performed before antiparasitic treatment. For the purpose of assessing clinical improvements, follow up times were grouped into time periods of 3–6 months, 7–9 months, 10–13 months and greater than 13 months. Peripheral blood eosinophil absolute counts tests were performed prior to steroid treatment to exclude the effects of steroids. Standard stool microscopy for *S*. *stercoralis* and other parasites was performed in all patients to exclude their effects on eosinophil counts. Peripheral blood eosinophil absolute counts of more than 0.5×10^9^/L were considered diagnostically to be eosinophilia. The patients avoided sparganum infection risk factors after antiparasitic treatments. Therefore, the possibility of reinfection decreased to a minimum level in the follow-up period.

### Radiological studies

All patients underwent conventional and contrast-enhanced MRI scans. The scanning equipment used in this study was a 3.0-T superconductive MRI system (GE Healthcare, Milwaukee, Wisconsin). The MRI scanning parameters included a 3-mm slice thickness and a 0–0.5-mm interval. The scanning sequences included an axial T2-weighted propeller sequence (4,600 ms/108 ms [repetition time (TR)/echo time (TE)]); an axial T1-weighted fluid-attenuated inversion-recovery (FLAIR) sequence (2,065 ms/22 ms [TR/TE]); and axial diffusion-weighted imaging (4,000 ms/64 ms [TR/TE], b = 1,000 s/mm^2^). The enhanced MRI included axial, sagittal, and coronal slices, a T1-weighted FLAIR sequence (2,065 ms/22 ms [TR/TE]), and the contrast agent gadopentetate dimeglumine (0.1–0.2 mmol/kg).

Our goal during the radiologic evaluations for both baseline and follow-up MRIs was to assess the motile and stable, chronic lesions. The radiological signs of motile lesions included aggregated ring-like enhancements, serpiginous enhancements, irregular enhancements, tunnel signs and migration signs. Aggregated ring-like enhancements, serpiginous enhancements and irregular enhancements were defined as ring-shaped or beaded enhancements, respectively, observed in an enhanced MRI[21]. A tunnel sign was defined as a path-like lesion with an enhanced edge that exhibited hypointensity on T1-weighted image (T1WI) and iso-/hyperintensity on T2WI^[^^9^^]^. The appearance of a new lesion in a follow-up MRI or a different location or shape of a lesion compared to the baseline MRI was considered to be a migrated lesions[13]. The stable, chronic lesions included white matter degeneration, cortical atrophy and ipsilateral ventricle dilation. All baseline and follow-up MRI images were reviewed by two experienced radiologists.

## Results

### Clinical findings

The demographic and baseline clinical findings from the 10 patients are shown in S1 Table. The patients came from seven provinces in China, including Heilongjiang, Hubei, Hunan, Jiangsu, Jiangxi, Fujian, and Yunnan provinces. During patient histories, all patients were found to reside in or have traveled to an endemic area and to have a history of ingesting uncooked frog or snake meat or of drinking contaminated water (contaminated water is defined as uncooked water in an endemic area) many times[1]. The primary symptoms in the baseline clinical findings included seizures in 7 patients, hemiparesis in 5 patients, headache in 5 patients, vomiting in 1 patient and altered mental status in 1 patient. The median duration of symptoms from onset to definite diagnosis was 45 months (range of 3 months to 188 months). Three patients showed peripheral blood eosinophil absolute counts of greater than 0.5×10^9^/L, and the other 7 patients were normal.

**Fig 1:**
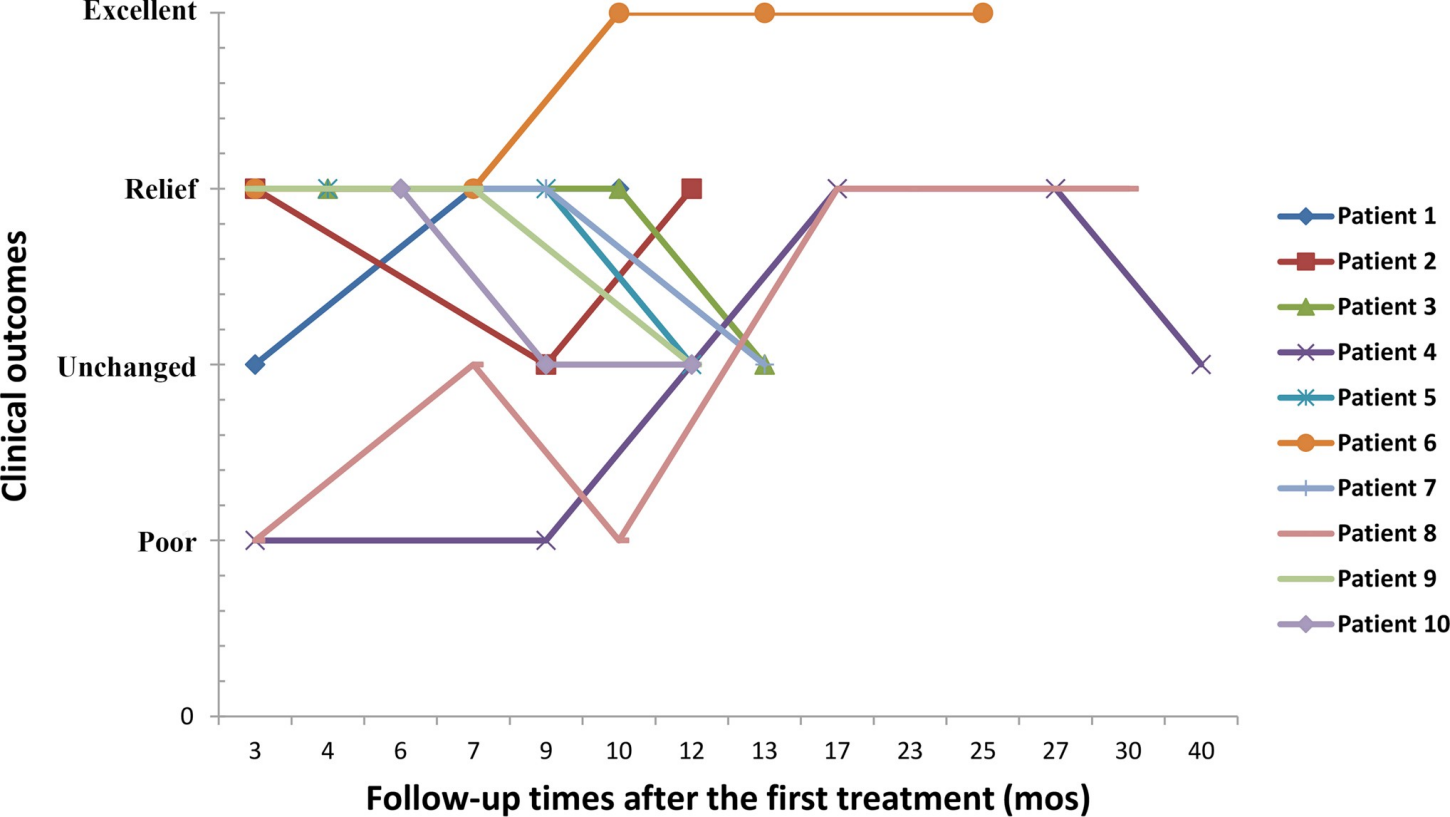
For the purpose of assessing clinical improvements, follow up times were grouped into time periods of 3–6 months, 7–9 months, 10–13 months and greater than 13 months. Most of the patients experienced an improving trend in clinical symptoms within 9 months after the first treatment, and a small number of patients with poor initial treatments presented with improved clinical symptoms during the 10–30 months after treatment.

The high-dose praziquantel therapy was well-tolerated in all patients. One patient received 1 treatment course, 5 received two treatment courses, 2 received three treatment courses, 1 received four treatment courses, and 1 received five treatment courses. Six patients (patients 1, 2, 3, 5, 6, and 7) presented with increased frequencies of seizures or the clinical symptoms of transient aggravation either during the course of treatment or 3 to 5 days after treatment. For each patient, at least 3 MRI scans and clinical follow-up studies were performed over periods ranging from 10 to 40 months. The follow-up information on clinical outcomes for the 10 patients are shown in Table 1 and Fig 1. The results of overall clinical outcomes showed that the symptoms were relieved without the appearance of new symptoms in 8 patients, the symptoms were eliminated completely in 1 patient, and the symptoms were aggravated in the 1^st^ through 3^rd^ follow-ups but were relieved in the last 2 follow-ups in 1 patient. The final clinical outcome of the patient with clinical symptom fluctuations was assessed as no change from baseline clinical symptoms. Two patients showed peripheral blood eosinophil absolute counts greater than 0.5×10^9^/L at follow-up.

**Table 1:** The proportions of each clinical outcome in the different follow-up interval groups.

Clinical outcomes	Follow-up interval			
	3–6 months	7–9 months	10–13 months	>13 months
Excellent	0%	0%	18%	14%
Relief	70%	56%	27%	72%
Unchanged	10%	33%	46%	14%
Poor	20%	11%	9%	0%

Clinical outcomes were divided into four groups including excellent, relief, unchanged and poor. Excellent (E): elimination of clinical symptoms; patients showed no symptoms.

Relief (R): clinical symptom relief and neurological deficit improvements without symptom elimination.

Unchanged (U): no change from latest follow-up in clinical symptom frequency and neurological deficits.

Poor (P): increased clinical symptom frequency and progressive neurological deficits or appearance of new symptoms.

### Radiological findings

**Fig 2:**
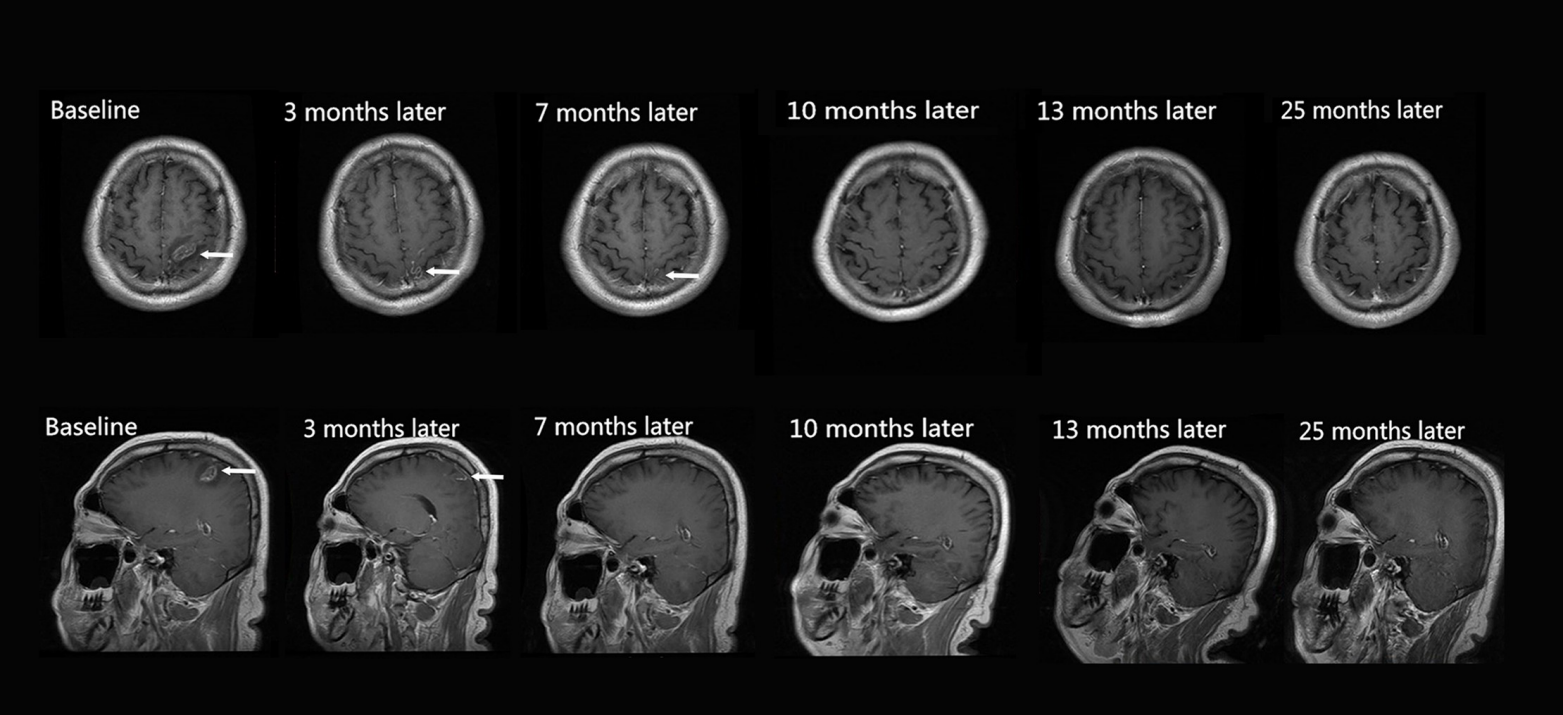
Axial and sagittal images of a 45-year-old male patient with a 3-month history of right hemiparesis. Case 6. An axial and sagittal T1 FLAIR image shows that there are serpiginous and irregular enhanced lesions in the left frontal and parietal lobes (white arrows) at baseline MRI. After 2 courses of therapy (10 months later) and follow-ups for 25 months, the left frontal and parietal lobe lesions disappeared completely, and no stable, chronic lesions occurred.

**Fig 3:**
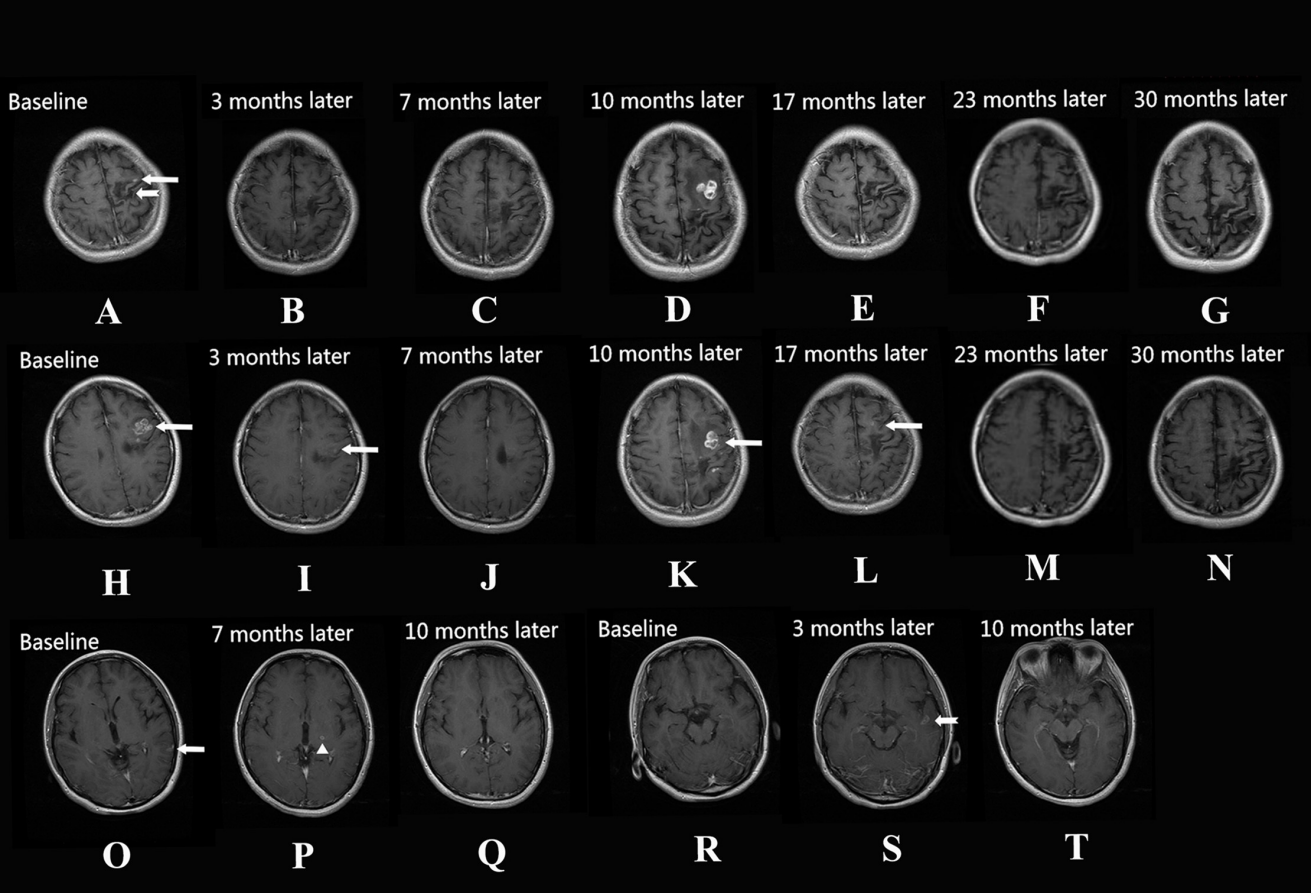
Axial images of a 22-year-old female patient with a 15-year history of seizures. Case 8. (A) (H). An axial T1 FLAIR image shows that there are aggregated ring-like, serpiginous and irregular enhanced lesions in the left frontal and parietal lobes (white arrows) at baseline MRI. White matter degeneration and cortical atrophy were found in the left frontal lobe (white open arrow) at baseline MRI. (B-G) (I-N). An axial T1 FLAIR image shows that after 2 courses of high-dose praziquantel therapy (7 months later), the left frontal lobe motile lesions disappeared. However, the initial aggregated ring-like and serpiginous enhanced lesions in left frontal lobe (white arrows) and parietal lobes reoccurred 10 months later. After 4 courses of therapy (17 months later), the extent of the left frontal lobe lesions (white arrow) are reduced significantly, and the left parietal lobe lesions disappeared. After 5 courses of therapy (23 months later) and follow-ups for 30 months, the left frontal lobe lesions disappeared completely, and white matter degeneration and cortical atrophy occurred in the corresponding area. (O-T) An axial FLAIR image shows that migrated lesions occurred in the left temporal lobe (3 months later) (white arrow and white open arrow) and the basal ganglia (7 months later) (white arrowhead) in follow-up MRI. After 2 courses of therapy (10 months later), the migrated lesions disappeared completely, and no long-standing lesions occurred.

**Fig 4:**
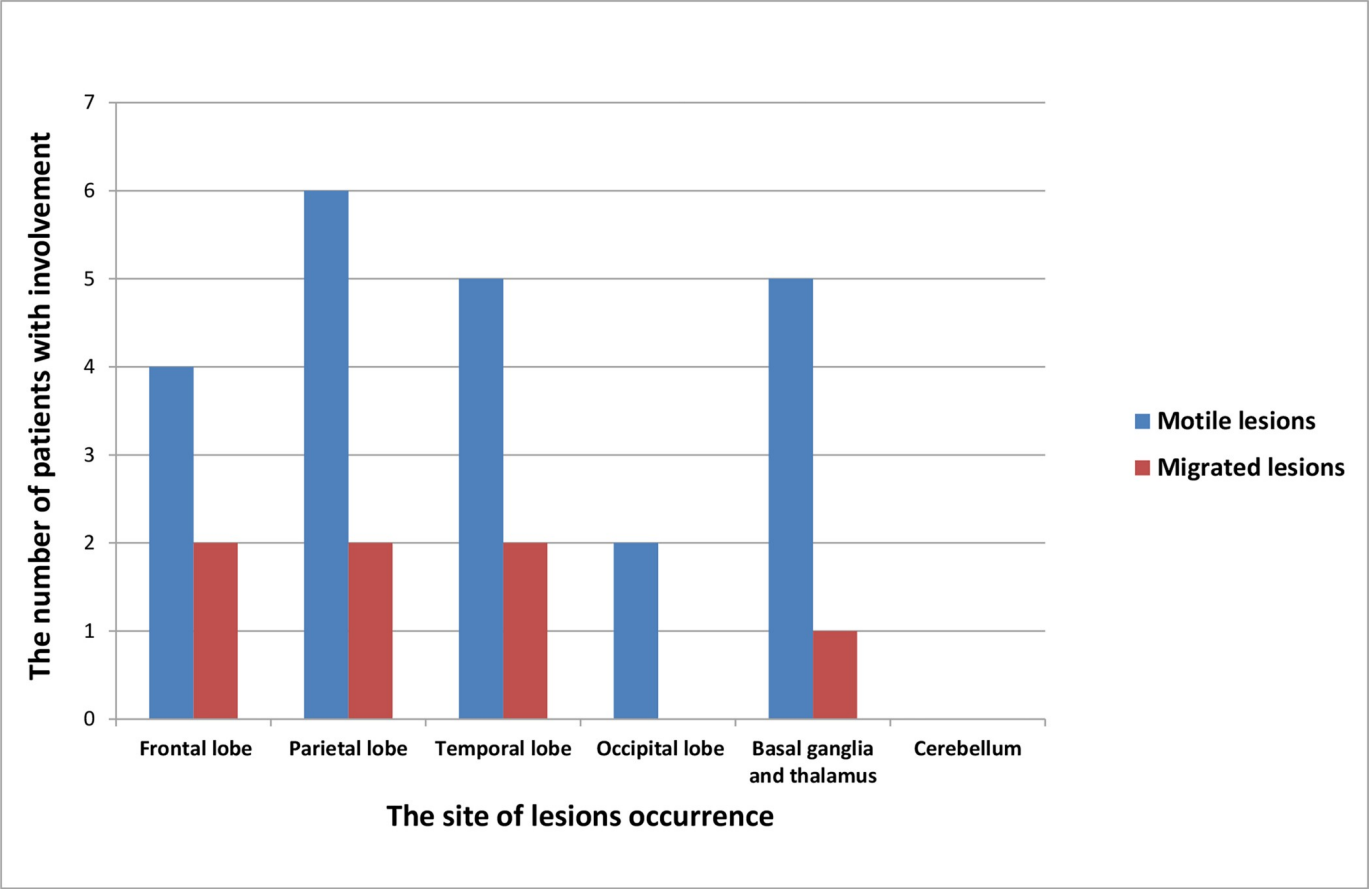
The distributions of motile and migrated lesions in brains. The motile and migrated lesions often involve the frontal, parietal, and temporal lobes and the basal ganglia but rarely involve the occipital lobe and the cerebellum.

The times taken for the radiological signs of motile lesions to disappear in follow-up MRIs from the 10 patients are summarized in Table 2. Ten patients showed motile lesions in their baseline MRIs, including aggregated ring-like enhancements (80%, 8/10 patients), serpiginous enhancements (70%, 7/10 patients), irregular enhancements (90%, 9/10 patients) and tunnel signs (50%, 5/10 patients). The patients had varying degrees of white matter degeneration (90%, 9/10 patients), cortical atrophy (80%, 8/10 patients) and ipsilateral ventricle dilation (80%, 8/10 patients) in their baseline MRIs. In the overall follow-up MRIs, 10 patients showed a disappearance of motile lesions, occurring at 3, 4, 6, 7, 9, 10, 12, 23, and 27 months after treatment, 80% (8/10 patients) of motile lesions were transformed into stable, chronic lesions, and 20% (2/10 patients) of motile lesions were eliminated completely (Fig 2 and Fig 3). All initial stable, chronic lesions were preserved without any changes in follow-up MRIs. Migration signs were found in 2 patients (patients 4 and 8), at 3, 7, 10, and 17 months after treatment. The distributions of the motile and migrated lesions from the 10 patients are shown in Fig 4.

**Table 2:** The proportions of each radiological sign that disappeared in different follow-up interval groups.

Radiological signs	Follow-up interval			
	3–6 months	7–9 months	10–13 months	>13 months
Aggregated ring-like enhancement	13%	37%	25%	25%
Serpiginous enhancement	29%	13%	29%	29%
Irregular enhancement	33%	11%	33%	23%
Tunnel sign	0%	40%	20%	40%

## Discussion

This study reports the effect of high-dose praziquantel treatment for cerebral sparganosis through clinical and MRI follow-up evaluations. Most patients achieved satisfactory treatment results, including the relief of clinical symptoms and the disappearance of enhancing lesions. In addition, we also observed that the time to clinical symptom relief did not match the time to enhanced lesion disappearance.

Cerebral sparganosis is a rare parasitic infection. Humans are intermediate hosts for *Spirometra mansoni*. The adult stage of *S*. *mansoni* primarily parasitizes the intestines of cats and dogs. The adult *S*. *mansoni* produces eggs in the intestines of cats and dogs that are then excreted in feces. Coracidia hatch from the eggs in contaminated water. Then, coracidia are ingested by *Cyclops*, which are the first intermediate hosts, and develop into procercoids, which are the first-stage *S*. *mansoni* larvae. Then, *Cyclops* is ingested by secondary intermediate hosts including frogs, snakes or humans, and procercoids develop into plerocercoid larvae, which are the second-stage larvae. Finally, the definitive hosts are infected by the second intermediate hosts, and plerocercoid larvae mature into adult *S*. *mansoni*[2]. Thus, the cycle of *S*. *mansoni* is completed.

Sparganum has the general life habits of a parasite: wandering and parasitism. The body of the larvae consists of bundles of longitudinal eosinophilic smooth muscle fibers. The anterior end of the larvae contains a characteristic groove called a scolex[9]. Proteolytic enzymes secreted by the scolex allow the sparganum to hydrolyze proteins and peptides, thus facilitating the penetration of loose connective tissue[22]. The smooth muscle bundles and proteolytic enzymes provide a strong contractile force and penetrability for wandering larvae. Previous animal studies have suggested that the route of sparganum migration into the brain involves the sparganum taking advantage of proteolytic enzymes to move through the gastrointestinal tract to the peritoneum or pleura. The sparganum then penetrates the subcutaneous tissue of the neck and passes through the foramen of the skull base to reach the brain[2].

The risk factors for *S*. *mansoni* infection include living in an epidemic area, eating raw/uncooked frog or snake meat, using raw frog or snake meat as a poultice on an open wound, and drinking unfiltered water contaminated with *copepods* harboring the parasite. The incubation period of sparganosis is not well defined, and the parasite is thought to live in the human body for decades[23]. In our study, an epidemiological history associated with sparganum, including ingesting frog or snake meat (70%, 7/10 patients) or drinking contaminated water (40%, 4/10 patients) many times, was an important factor in the diagnosis of patients with this condition. Several scholars have suggested that children and young people are more prone to cerebral sparganosis, particularly those 5 to 30 years old[2, 24, 25]. In this study, the mean age at diagnosis was 20.3 years old. Possible reasons for this vulnerability include the following: the immature development of the immune system and the blood-brain barrier can allow the larvae to survive in the human body longer and provide easier access to the nervous system; and young people or children usually play in pools or brooks, which can increase the chances of contacting parasite-contaminated water.

Cerebral sparganosis is the most serious human sparganosis complication. Clinical manifestations of cerebral sparganosis vary and often depend on the cerebral region that is infected[18, 26, 27]. Because of the more widespread motile or chronic lesions in the white matter and the excessive electrical discharge of brain cells caused by live larvae migrating in the cortex or subcortical areas, seizures are the most commonly observed symptom[2]. In our study, 70% (7/10 patients) of the patients presented with seizures. In addition, our patients showed several nonspecific clinical symptoms including headache (50%, 5/10 patients), vomiting (10%, 1/10 patient), hemiparesis (50%, 5/10 patients) and altered mental status (10%, 1/10 patient). Based on these nonspecific clinical symptoms, it is very difficult to make a diagnosis. However, based on our results, we believe that, if these nonspecific clinical symptoms change frequently in a short time, this could be a useful clue indicating wandering live larvae.

The parasite survival status, parasite-induced secondary acute and chronic inflammations, and the responses after treatment are all associated with the pathological basis of imaging findings[28].The aggregated ring-like enhancements may reflect the cross-sections of small abscesses. The characteristic edema signal may reflect local inflammation. The signs of serpiginous enhancements and irregular enhancements may reflect the slender and twisted forms of the worm’s body. The tunnel signs can be visualized between the primary lesion and the surrounding lesions, suggesting the migration of a live worm and the formation of eosinophilic granulomas. Sometimes, multiple lesions around the worm body suggest the different stages of eosinophilic granulomas. The migration sign is often defined as changes in new lesion locations, shapes, and styles during follow-up MRIs, relative to the initial lesions observed in the baseline MRI, which strongly suggests the movement of live worm larvae[9]. The above radiological signs also indicate that the lesions are motile lesions. Moreover, stable, chronic lesions are related to chronic inflammation[9]. In our results, we noticed that the time to the disappearance of the radiological signs of motile lesions usually lagged behind the time to clinical symptom improvements. Meanwhile, we also observed that the radiological outcomes of motile lesions varied with different courses of treatment. In addition, in our results, most of the motile lesions transformed into stable, chronic lesions after treatment, which often suggests that the clinical symptoms will improve but will not be eliminated. If the follow-up MRI shows that no stable, chronic lesions occur after the motile lesions disappear, this often indicates a good prognosis. We also observed that the frontal, parietal, and temporal lobes and the basal ganglia were often infected with motile and migrated lesions but the occipital lobe and the cerebellum did not present the same trend. Moreover, based on the results of the distribution of the migrated lesions, we noticed that the migrated lesions usually occurred in close proximity to the initial lesion. These results are in accordance with those of a previous study[13]. For migrated lesions, we recommend that, when migration signs appear in a follow-up MRI, the choice of a sequential treatment between praziquantel treatments and surgical resection should be based on the severity, location and number of lesions.

Peripheral blood eosinophilia suggests the presence of a parasitic infection. However, only three patients (30%, 3/10 patients) showed peripheral eosinophil absolute counts >0.5×10^9^/L in the baseline tests. The follow-up results showed that the peripheral eosinophil absolute counts were often <0.5×10^9^/L, regardless of clinical outcomes. Therefore, we believe that there were no direct relationships between peripheral eosinophil absolute counts and clinical outcomes. In contrast, many studies have shown that ELISAs of the serum and cerebral spinal fluid (CSF) have a high sensitivity for the diagnosis of cerebral sparganosis[13, 18, 24]. Several studies have reported a sensitivity between 80% and 100%[3, 24]. Therefore, cerebral sparganosis should be diagnosed based on epidemiological history, clinical manifestations, ELISA testing and radiological findings.

The sparganum cannot reproduce, but it has a long lifespan (it can survive for up to 35 years) in the human body[24]. Therefore, surgical resection or stereotactic surgery is recommended as an effective treatment. However, there is currently no specific treatment for inoperable patients. The pharmacological and toxicological effects of praziquantel on sparganum include two aspects. First, praziquantel can cause spastic paralysis and dissolve the smooth muscle bundles in the worm. Second, praziquantel can result in epidermal erosion by damaging the skin of the worms, which then exposes the surface antigens of the parasite[19]. Moreover, the pharmacodynamics of praziquantel therapy for cerebral sparganosis also has two characteristics. On the one hand, praziquantel reaches a peak plasma concentration 2 hours after administration and decreases rapidly in a short time. On the other hand, although praziquantel penetrates the CSF quickly during the biological half-life of the drug, it enters parasites slowly. Due to the barriers between human bodily fluids and parasites, it is necessary to increase the drug concentration 7–10 times in the CSF or brain parenchyma to effectively kill the parasites[17, 19]. Previous studies have shown that conventional-dose praziquantel therapy (120–200 mg/kg divided over 2–4 days) is often considered to be less effective [2, 3, 14, 24]. One study reported that 3 patients experienced migrated lesions among 5 patients who received conventional-dose praziquantel therapy, and only one patient presented with clinical symptom improvement among the 5 patients[14]. However, there have been several case reports that have shown that high-dose praziquantel therapy (500–750 mg/kg divided over 7–10 days) is effective for cerebral sparganosis[17, 18]. One case report discussed a single adult patient who received high-dose praziquantel therapy, was followed for 3 years and presented with significantly improved clinical symptoms and MRI findings[17]. Therefore, based on the pharmacokinetics of praziquantel and the viewpoints of Gonzenbach RR et al., we believe that high doses and multiple doses (daily praziquantel at a dose of 75 mg per kilogram of body weight, which was administered in three divided doses) over a relatively long course (10 days per course) of treatment could be more conducive to allow praziquantel to cross the blood-brain barrier and enter parasites to achieve pharmacological effects[17]. Even at this dose, praziquantel is generally well tolerated, as supported by the treatment of other parasites[29].

In our study, 8 patients (80%, 8/10 patients) showed gradual improvements after multiple high-doses of praziquantel treatments. Compared with baseline data, the decreased seizure frequency was found in 6 patients (75%, 6/8 patients) and neurological deficit improved such as limb weakness relieving, and level of muscle power increasing was observed in 4 patients (50%, 4/8 patients) at end of follow-up. One patient (10%, 1/10 patient) recovered with normal life after treatment accompanied by the elimination of clinical symptoms. Two of the above 9 patients showed motile lesions that disappeared completely in MRI tests. In addition, one patient (10%, 1/10 patient) experienced fluctuations in clinical symptoms and radiological findings. However, relative to baseline data, the patient did not show significant decreased seizure frequency or mental status improvements. We speculate that these responses could be related to the incomplete immune system development at the young age of onset and the abnormal immune response caused by a longer infection period. Moreover, during the process of treatment or the post treatment period, we found that 6 patients (60%, 6/10 patients) showed clinical symptoms of transient aggravation. We observed that the prognoses of these patients were often improved at follow-up; 1 patient recovered, and 5 patients obtained relief. We speculate that this type of post treatment reaction may be associated with the disintegration of the worm’s body caused by the drug effects, which usually associated with the good long-term prognosis. Furthermore, we found that the clinical improvements primarily occurred within 9 months after the first treatment for most patients. However, the improvements of the few patients with poor initial treatment outcomes occurred during 10–30 months.

Finally, we acknowledge some limitations of the present study. First, the follow-up intervals of this study are relatively long, which makes more subtle therapeutic changes difficult to observe. Second, because of the retrospective nature of this study, is no comparative trial was designed, although previous studies that have reported conventional-dose praziquantel therapy for cerebral sparganosis have been applied as a comparison. Third, also due to limitations of a retrospective study, the baseline and follow-up information for each patient cannot cover all required examination items, such as head CT scan and specific antibody detection in the CSF by ELISA. Further comparative studies will be conducted in the future. This work provides evidence to suggest that high-dose praziquantel therapy for cerebral sparganosis is effective and is a promising alternative for the treatment of cerebral sparganosis, particularly for inoperable patients. Peripheral eosinophil absolute counts cannot be used as an effective prognosis indicator. The radiological outcomes of motile lesions are an important indicator of the process of the treatment. Motile lesions will generally become stable, chronic lesions after treatment, and timely and effective treatment may eliminate chronic lesions in the brain. Because MRI improvements may lag behind clinical symptom improvements, it is necessary to perform MRI follow-ups 1–3 times after clinical symptoms show improvements. We hope that our findings may bring some inspiration to the treatment and management of cerebral sparganosis.

## Supporting information

S1 Table Demographic and baseline clinical findings from 10 patients. (DOCX)
