# Supplementary material for: Follow-up study of high-dose praziquantel therapy for cerebral sparganosis
Source: PLoS Negl Trop Dis. 2019 Jan 14;13(1):e0007018. doi: 10.1371/journal.pntd.0007018 (PMC6331082; doi:10.1371/journal.pntd.0007018)
Supplement: S1 Table — (DOCX) [file pntd.0007018.s001.docx]

S1 Table Demographic and baseline clinical findings from 10 patients

| Patients | Age  /sex (yrs) | Ingestion history | Clinical manifestations | Duration of symptoms (mos) | Serum ELISA test | Eosinophil absolute counts  (×10^9^/L) | Treatment of high-dose praziquantel (courses) |
| --- | --- | --- | --- | --- | --- | --- | --- |
| 1 | M/25 | Eating raw frog meat | Seizure, headache | 138 | (＋) | 0.054 | 2 |
| 2 | M/17 | Living in rural areas around a lake and eating raw frog meat | Seizure, headache, left hemiparesis | 60 | (＋) | 0.172 | 2 |
| 3 | M/26 | Eating raw frog and snake meat | Seizure, headache, left hemiparesis | 150 | (＋) | 0.069 | 3 |
| 4 | M/7 | Playing in the brook and drinking contaminated water | Headache, vomiting | 30 | (＋) | 0.094 | 4 |
| 5 | F/15 | Swimming in the lake and drinking contaminated water | Seizure, headache, right hemiparesis | 11 | (＋) | 0.049 | 1 |
| 6 | M/45 | Eating raw frog meat and drinking contaminated water | Right hemiparesis | 3 | (＋) | 0.541 | 2 |
| 7 | F/20 | Eating raw frog meat | Seizure | 5 | (＋) | 0.573 | 2 |
| 8 | F/22 | Eating raw frog meat | Seizure with altered mental status | 188 | (＋) | 0.221 | 5 |
| 9 | M/8 | Living in rural areas around a river and drinking contaminated water | Right hemiparesis | 72 | (＋) | 0.613 | 3 |
| 10 | M/19 | Eating raw frog meat | Seizure | 12 | (＋) | 0.072 | 2 |

ELISA: enzyme-linked immunosorbent assay

(＋): positive
